# A new immunological paradigm for diseases: the protein-homeostasis-system hypothesis

**DOI:** 10.3389/fimmu.2026.1944127

**Published:** 2026-09-22

**Authors:** Kyung-Yil Lee

**Affiliations:** 1 The Catholic University of Korea College of Medicine, Seoul, Republic of Korea; 2 Junglock Biomedical Institute, Deajeon, Republic of Korea

**Keywords:** etiological substances, etiology, oncogenesis, organicism, pathophysiology, protein-homeostasis-system hypothesis

## Abstract

Contemporary immunology models are based primarily on reductionist concepts. However, they face limitations in reconciling clinical discordances such as the ‘viral load paradox’ in severe infections and the teleological enigma of the ‘autoimmune paradox’. To bridge these systemic conceptual gaps, this paper proposes the protein-homeostasis-system (PHS) hypothesis, a novel paradigm based on organicism that views the living organism as a unified, self-regulating biosystem. It is asserted that all diseases, including autoimmune disorders and malignancy, are triggered by specific etiological substances—ranging from toxic elements and monoamines to pathogenic peptides and proteins, many of which originate from self-cells injured by infection, traumas, and cytokine storm. Within this overarching framework, the immune system’s role is fundamentally redefined from an adversarial defense mechanism into a metabolic and regulatory management utility for cell protection and systemic homeostasis. Governed by the PHS, T cells and B cells regulate the systemic load of pathogenic peptides and proteins, while the innate immune system, including prions and amyloid proteins, manages non-protein materials. Acute clinical phenomena like cytokine storms are reinterpreted not as immune system autotoxicity, but as the desperate hyper-activation of non-specific immune clones responding to an overwhelming endogenous etiological load. This holistic perspective provides a robust translational rationale for the early, preemptive clinical use of systemic immunomodulators, including corticosteroids, intravenous immunoglobulins and biologics, to disrupt pathological feedback loops and restore rapid immunological stabilization, and views cancer as an independent biosystem’s survival strategy.

## 1 Introduction

Human beings suffer from a myriad of diseases. Biomedical research has advanced significantly toward therapeutic strategies by identifying the deficiency or excess of specific substances or dysfunctions in regulatory mechanisms; however, the fundamental etiology and pathophysiology of many diseases, including infectious diseases, autoimmune disorders, cancers, and idiopathic conditions, remain elusive.

Inductive reasoning has been accepted for important propositions in scientific research without direct proof, including the existence of dark matter and entropy in physics, the theory of plate tectonics in geology, and the theory of evolution in biology (1, 2). Although multicellular organisms, including human species, seem to have evolved from unicellular origins, it is difficult to clearly define life itself in scientific terms (3, 4). All biological phenomena are controlled by multiple systems within the organism. Researchers in biomedicine tend to think that understanding the etiology or pathogenesis of diseases is difficult because they expect disease mechanisms to be diverse and assume that they lack a suitably advanced approach to finely dissect those mechanisms. In addition, genetic and environmental factors are associated with many diseases.

From a molecular biological perspective, the essence of disease is the destruction or dysfunction of organ cells, and numerous inflammation-inducing substances exist within affected cells including damage-associated molecular patterns (DAMPs), pathogen-associated molecular patterns (PAMPs), biochemicals, and pathogenic peptides/proteins. Current immunology is based on a dichotomous way of thinking that divides immune system into adaptive and innate immune components, prioritizing the body’s defensive functions. Therefore, it is difficult to explain the pathogenesis of disease using established immunological dogmas. For disease recovery, all immune cells and immune proteins are involved in mechanisms to eliminate the causative agents and replace damaged cells with new cells, and mutual communication between immune cells as an immune network is required. This communication takes place through cytokines and their receptors expressed on the cells in the immune network. Although biomedical research predominantly takes a reductionist approach, organicism and reductionism coexist as fundamental scientific concepts about life as a biosystem, and there is a growing agreement that combining these two concepts is needed to solve problems in biomedicine including immunology (5–7).

To date, few unified theories have been proposed to comprehensively explain the etiology and pathophysiology of diseases, largely because the current paradigm assumes the presence of unidentified etiological substances at disease onset and the limitations of the current immunological paradigms used to explain the pathophysiology of each disease.

The author previously introduced the protein-homeostasis-system (PHS) hypothesis to integrate the etiology and pathophysiology of diseases, including infectious diseases, organ-specific disorders, autoimmune and allergic disorders, genetic diseases, and cancer (8–13). The hypothesis adheres to the principle of scientific causality: every disease has etiological or inflammation-inducing substances, the immune system controls those substances under the regulation of the PHS, and the PHS partially compensates for protein deficiencies within cells and the host. Although the hypothesis is grounded in organicism, which views organisms, including humans, as unified biosystems controlled by the PHS, it offers a macroscopic perspective of disease and suggests the use of reductionist methods to validate it. This paper reviews currently unresolved issues in diseases, provides new insights into disease pathophysiology, and underscores the need for holistic approaches in medical interventions.

## 2 Nature of diseases

Disease can be defined as a phenomenon that emerges during the battle between pathogenic agents and a host’s defense system against them. Histopathological findings from each disease show the deposition of immune cells and/or immune proteins with or without the destruction of cells. In this metaphor, inflammatory lesions can be regarded as battlefields between pathogenic agents and host defenses. A variety of immune system components act as combatants on the battlefield and are recruited through systemic circulation in the host. If the battle is won, the battlefield is restored, and the patient recovers from the disease. However, if the enemy is not eradicated, the battle can continue for a long time, resulting in chronic illness, and if too many enemies to control at once are present, the host can be ruined. It is an important notion that the battlefields contain enemies, combatants, and corpses from both forces.

Individual people with any disease exhibit different clinical presentations, progressions, and prognoses. For example, COVID-19 is a self-limited disease in most people, but some patients, even healthy immune-competent ones, experience pneumonia and acute respiratory distress syndrome (ARDS) in the acute phase and can experience chronic disease or other organ dysfunctions after an acute episode (14). This phenomenon is also observed with other respiratory infections such as influenza and mycoplasma infection (15). In acute infection-associated immune-mediated disorders, such as acute rhematic fever, Kawasaki disease (KD), and multisystem inflammatory syndrome in childhood (MIS-C), and in chronic immune-mediated diseases, such as juvenile idiopathic arthritis (JIA), rheumatoid arthritis (RA), and systemic lupus erythematosus (SLE), patients show varied clinical manifestations and different involved organs. Also, patients with cancer, Alzheimer’s disease, or genetic disorders caused by a single gene defect show disease progressions, treatment responses, and prognoses that differ among individuals. Thus, it can be expressed metaphorically that the distinct phenotypes of disease are determined by both the types or kinds of enemies and the places they invade within the host, and that individual variations in the clinical course of a single disease are determined by the number and types of enemies and the strength and ability of an individual’s combatants against them.

A variety of cells in the tissues and organs of the host express unique receptors and produce and use their own proteins and peptides. The functions of each cell are predetermined by so called “cell fate”, including cells in development (16). Similarly, it is acceptable that the roles of each combatant in the innate immune system and the adaptive immune system are predetermined. In other words, the basic roles of each combatant, including those mobilized to battlefields against diverse enemies in all diseases, are the same.

Systemic homeostasis in multicellular organisms is achieved through the homeostasis of all the cells in all the tissues and organs (17). Special substances, such as hormones produced by cells in a tissue or an organ, are used by cells in other organs, and systemic communication across cells occurs through receptors expressed on the cells (18). Binding between biologically active substances and the receptors of target cells activates signal transduction pathways and induces various cellular responses, including the activation of genes for corresponding proteins, the secretion of preserved intracellular materials, and the induction of apoptosis in targeted cells (19). Therefore, this process can occur across cells in immune networks. Specific cells in tissues or organs express receptors for elements, monoamines, neuropeptides, peptides and proteins, and other biochemicals (hormones, vitamins, fatty acids, etc.). Deficiency of a single substance, such as vitamin C leading to scurvy, and excess of nutrients resulting in metabolic syndrome both demonstrate the critical link between biochemical balance and systemic homeostasis. Furthermore, certain substances that are harmful to cells can affect targeted cells that have an affinity to them and induce cellular responses that result in inflammation and cell injury. However, such toxic substances cannot affect cells that do not have affirmative receptors in the host. For example, current anticancer drugs target receptors expressed primarily on or in cancer cells to control the target cancer cells. Both synthetic drugs, including monoclonal antibodies and chemicals (20), and complex natural toxins such as venoms—which consist of diverse peptides, proteins, and biochemicals—exert their effects by binding to specific receptors on the target cells (21). Animal models of chronic or autoimmune diseases, such as experimental encephalitis or SLE, have revealed that certain cell contents can induce inflammation (22). Also, transient or long-term appearance of autoantibodies to self-proteins/peptides can be detected in health people as well as patients with diseases, and DAMPs can induce inflammation as well as PAMPs. Therefore, the destruction or dysfunction of target cells in organs is a fundamental mechanism in the pathophysiology of disease, and substances that originate in injured cells can affect cells in same or other tissues and organs, and thereby, the entire host.

## 3 Etiology of diseases

Although all diseases have an etiology, many etiologies are described as “idiopathic,” “essential,” or “primary,” which just means “unknown,”. Diseases with clearly identified causative agents or events include infectious diseases, allergic conditions, and certain genetic disorders, but the pathogenesis of cell injuries in those diseases remain unknown. Causal pathogens, causal allergens, and dysfunctional proteins themselves are not toxins that can directly cause cell injury in organs. For instance, analyses of the extensive destructive lung lesions observed in severe cases of COVID-19, influenza, and mycoplasma infection demonstrate that the lesions contain few intact pathogens, with only occasionally positive results in polymerase chain reactions or anti-pathogen antibody tests (23, 24). Animal studies demonstrated that intraperitoneal or peroral inoculation with the influenza virus or *Mycoplasma species* produce similar temporal appearance of pneumonia compared to intranasal inoculation with a few pathogens in the lungs (25), and T cell-deficient animals show less severe lung injury than immunocompetent controls (26), suggesting that pneumonia-eliciting substances in these infections are not pathogens but those associated with T cell-activation. Similarly, few studies have revealed crude allergens as the causes of pathologic lesions in allergic diseases such as asthma and atopic dermatitis. Pathogens and allergens are complex materials, and the directly toxic or inflammation-inducing substances that cause target-cell injury are smaller molecules, not the pathogens or allergens themselves. In most genetic diseases, the affected proteins have multiple or unknown functions, and the mode of action of the protein is associated with other proteins or biochemicals *in vivo*. As seen in diseases such as Huntington’s disease or Duchene muscular dystrophy, the affected proteins, huntingtin and dystrophin, respectively, often function as central nodes in complex biological networks (27, 28).

In science, *etiology* refers to specified substances that are directly associated with disease onset. In the blood, tissues, and cells of human beings, new types of substances are continually being discovered through modern omics technologies. The human genome contains approximately 20,000 protein-coding genes, but after post-translational modification and splicing, the number of actual protein species (proteoforms) present in blood and tissues is estimated to exceed one million, and clinically only 2–3 hundred proteins in plasma are used in disease diagnosis and therapeutic monitoring (29). As for peptides, over 10 thousand types are identified, including fragments generated during protein degradation in proteasomes and self-peptides presented to major histocompatibility complexes (MHCs) (30, 31). The PHS hypothesis accounts for small-molecular-weight materials as “non-protein substances.” Only 25 to 30 elements are essential for human bodies, and they are categorized as major (Ca, Mg, K, Na, etc.) and trace (Fe, Zn, Cu, Se, etc.) elements. Some elements determine the structure of proteins (metalloproteins) or act as enzyme activity switches; imbalances in their concentrations (e.g., excess copper or iron) lead directly to protein misfolding (32, 33). Monoamines and neurotransmitters consist of 50 to 100 major molecules, including acetylcholine, dopamine, gamma-aminobutyric acid, and neuropeptides, along with their metabolites. Although they are very small, they induce powerful production signals and must therefore be strictly controlled by systems, especially within the central nervous system (CNS) (34). There are more than 200,000 human metabolites, including lipids, amino acids, nucleic acids, and sugars (35). Lipids are extremely diverse and account for the largest proportion of metabolites (36). In addition, diverse chemicals such as pollutants, food preservatives or additives, drugs, and biochemicals derived from pathogens, parasites, insects, plants, and animals can all occasionally be contained.

Organisms, including humans, have distinct biological subsystems that control all those substances, and among them, the immune system might control substances that are pathogenic to self-cells. The higher-level system that integrates all the substances and subsystems to maintain overall homeostasis is named the PHS, which is a different term from the proteostasis which refers only to protein homeostasis within cells.

Etiological substances of diseases are defined in the PHS hypothesis as toxic and bioactive substances that bind to affinitive receptors on target cells. The binding process affects the target cells’ response inducing recruitment of corresponding immune components, and then inflammation processes begin to protect the affected cells. On occasion, immune responses against excessive loads of etiologic substances together with marked activation of immune components with cytokine imbalances could be responsible for target cell injuries in diseases.

The etiological substances of diseases could be classified as external (or exogenous) and internal (or endogenous) in origin. For example, the former includes small toxic substances derived from pathogens, allergens, environmental toxins, the products of dysbiotic microbiota, and ingested foods, and the latter includes materials derived from self-cells such as pathogenic peptides/proteins, DAMPs, and other biochemicals.

## 4 Pathophysiology of diseases

### 4.1 Brief review and unresolved issues in current immunological models

Current immunological paradigms, while insightful, can be seen as mired in dichotomous logic because they posit an innate immune system (granulocytes, monocytes and macrophages, natural killer (NK) cells, and mast cells) and an adaptive immune system (T cells and B cells). However, all the components of the immune system work not only against infectious diseases but also against nearly all pathologies, including wound healing (tissue regeneration), tissue graft rejection, allergies and autoimmune diseases, specific organ diseases, and cancer. Immunological specificity and memory are known as a characteristic of adaptive immune cells. However, innate immune cells such as NK cells and monocytes also have an immunological memory called *trained immunity* (37, 38). Toll-like receptors (TLRs) are thought to be expressed on innate immune cells mainly to control PAMPs (39), but recent studies have found that T cells also express TLRs and use MyD88 in signal transduction pathways (40, 41), suggesting that T cells could control their activity by identifying danger signals. The idea that the immune system has an inherently defensive mechanism to protect the host through an innate capacity to distinguish between benign and toxic substances, or self and non-self, has been accepted based on organicism. The ‘self and non-self’ paradigm has also been changed since the discovery that DAMPs induce similar immune responses through pattern-recognition receptors as well as PAMPs (42, 43).

In infectious diseases, the current paradigm explains that the innate immune system works against infectious insults first, and then the adaptive immune system begins working later, and the pathogen itself is often cited as the cause of cell injury, especially in viral diseases that are held to exert virus-induced cytopathy. However, toxic or inflammation-inducing substances in viral infections can be derived from virus-infected cells, not the virus itself (9). The idea that the immune system, in addition to its defensive mechanism, sometimes attacks self-cells or causes harm to the host through abnormal or excessive immune responses has been accepted; self-cell reactive T cells and B cells (autoantibodies) in autoimmune diseases and overacting cytokines and intracellular materials derived from mast cells/basophils or eosinophils in allergies are associated with cell injury (44, 45). However, the reasons and mechanisms for those phenomena remain to be further evaluated.

In tumor immunology, it is believed that some cancer cells are recognized as “non-self,” and that the immune system of the host works against those using the same mechanisms it uses against pathogens or foreign grafts (46). On the other hand, most cancer cells express self-MHCs and receptors for communicating with host cells, including macrophages and stromal cells, within tumor microenvironment (TME) to ensure their survival and homeostasis (47), making them unlike invading pathogens. Furthermore, phenomena observed in *in vitro* and *ex vivo* studies, such as NK cells and cytotoxic T cells killing tumor cells (or virus-infected cells), do always not occur within human bodies (48).

For tissue repair (or wound healing), even in sterile surgical operation, immune cells in tissues such as mast cells and circulating immune cells, including platelets, neutrophils, monocytes (macrophages), T cells, and immune proteins, are mandatory to work under the integrated control of the PHS (49–51). Innate lymphoid cells (ILCs) are characterized as tissue-resident lymphocytes lacking rearranged antigen receptors, and their subtypes—ILC1, ILC2, and ILC3—precisely mirror the cytokine profiles of helper T cell subsets (Th1, Th2, and Th17, respectively) but operate with immediate, antigen-independent kinetics at barrier surfaces (52). These cell subsets express specialized receptors for alarmins or DAMPs, and various biochemicals liberated from injured cells, and their activation is observed in any diseases with tissue destruction, suggesting that this cell population might be not merely a ‘defense cell’ but play a role in repairing tissue damage by immediately recognizing cell destruction and regulating harmful or necessary substances for maintaining tissue homeostasis (53).

Based on insights from organicism, it is widely accepted that T cells, including regulatory T cells, play a central role in maintaining immune homeostasis in disordered conditions (54). The Th1/Th2 paradigm and balance of T cell subsets (Th1, Th2, Th17, and Treg) explain disease pathogenesis as broken balances of T cell subsets (55, 56). T cell studies have revealed that any subset of T cells, including Th1, Th2, Treg, and cytotoxic T cells, requires peptides to function (or be activated). T cell receptors (TCRs) on T cell subsets identify peptides within self-MHCs expressed on antigen processing cells (APCs) or other host cells, and T cells recognize both self-MHCs and peptide antigens within MHCs, which is called *MHC restriction* (57, 58). MHC class I and class II molecules exhibit polymorphism among individuals, and MHC class I molecules are expressed on nearly all cells within tissues and organs and play a critical role in the success of organ transplantation (59). Importantly, peptides are found within the cells of all organisms on earth, including bacteria, yeast, plants, insects, and mammals, indicating that they are essential molecules within cells and play a crucial role in maintaining systemic homeostasis in organisms (60, 61).

Looking at these phenomena from another angle, it is acceptable that MHCs function as peptide-binding receptors of self-cells and that T cells could distinguish peptides that are essential for the organism from pathogenic peptides derived from animals and other people, as well as peptides from self-cells that are blocked from release in a steady state. During T cell development in the thymus, T cell clones that recognize self-cells are deleted through so called *negative selection* (62), and it could be alternatively explained that T cell clones recognize essential peptides expressed on the MHCs of various types of self-cells in the thymus. Thus, those clones might be needless for the future protection of self-cells, so only T cell clones that can recognize pathogenic peptides are generated. It is an important notion that individuals with polymorphic MHCs might use different essential peptides based on their unique genetic profiles. In the evolutional view, if cells in individuals used same essential peptides, all individuals in the human species could be affected when toxic, cross-reactive peptides are encountered. Studies on the association between MHCs and diseases, including human leukocyte antigen B27 and ankylosing spondyloarthritis, have shown that it can be explained in part through this assumption (63).

In an MHC-restricted immune response, a specific peptide antigen presented by (i.e., bound in) MHC molecules leads to the generation of antigen-specific T cell clones through TCR-associated signaling pathways (64). The activated T cell clones by this process secrete cytokines and biochemicals that might be preserved in the granules of T cells. For example, certain CD4+ T cell subsets secrete IL-4, IL-6, IL-12, or other cytokines for the maintaining of helper T cell subset and production of specific antibodies by B cells (65), and certain cytotoxic CD8+ T cell subsets secrete cytotoxic materials, including perforins or granzymes, preserved within them to control their target cells (66). In a classical MHC-restricted specific immune response, the peptides are a part of proteins derived from pathogens or toxic complex materials processed by APCs. It is plausible that those peptides might have pathogenic potential and must be tightly regulated or removed to prevent them from damaging host cells. Therefore, T cell–B cell interactions might be more closely related in the immune network, and T cell activation is more tightly regulated by co-stimulatory and inhibitory receptors such as CD28, CTLA-4, and PD-1 (67, 68) due to the essential role played by peptides in the host.

On the other hand, T cells can be rapidly activated by superantigens, mitogens, drugs, and antibodies targeting TCRs both *in vitro* and *in vivo*, independent of MHCs (69–72). Experimental evidence also suggests that free peptide antigens *in vitro* and *in vivo* can bypass the conventional processing pathway to directly interact with TCRs, potentially leading to T cell proliferation or peripheral anergy (73, 74), suggesting that free peptides are also associated with T cell activation. Research has revealed that T cells recognize not only peptides but also other biochemicals, which are bound on the receptors such as CD1 for lipid antigens (75), and that γδ T cells can recognize biochemicals derived from pathogens such as toxic glycoproteins without MHC restriction (76). T cell subsets in intestines capable of communicating with microbiota are selected or restricted by MR1, a monomorphic MHC class I-related molecule that is markedly conserved in diverse mammalian species (77). Therefore, T cell responses could be categorized as MHC-restricted and non-MHC-restricted. T cells in the MHC-restricted pathway specifically recognize peptides with TCRs that are determined by the recombination of immune genes (V-D-J) as well as B cell receptors (BCRs) (78), whereas T cells in the non-MHC-restricted pathway might be involved in nonspecific, more broadly reactive (cross-reactive) and temporally rapid immune responses. T cell-absent mammals as well human neonates show normal embryogenesis with intact biological functions at birth (79). Unlike T cells, macrophage-lineage cells are indispensable for embryogenesis and structural development; their absence leads to severe developmental failure or lethality (80). Moreover, T cell subsets exhibit significant plasticity in various disordered conditions (81), thus the distribution of T cell subsets differs among individuals with the same disease and indeed changes during disease processes, suggesting that T cells might have limited functions in maintaining systemic biological or immune homeostasis. Considering their high plasticity and sensitivity to the biochemical milieu (82), T cells could be characterized as a biological readout or a dependent variable in disordered conditions. Thus, their activation and subset distribution could be dictated by the etiologic substances, mainly pathogenic peptides, as the true independent variables in biological phenomena such as disease processes, transplantation rejection, and tissue repair. The peptides identified by TCRs have variable sizes depending on the subset of T cells, but smaller peptides, including monoamine derivatives and neuropeptides, and larger peptides as small proteins cannot be controlled by T cells.

Patients with T cell deficiencies show a more critical prognosis than those with B cell deficiencies (83), and the proportion of T cells in the blood is greater than that of B cells, indicating that the control of pathogenic peptides by T cells might be more critical for systemic biological homeostasis than that by B cells. Taking the evidence together, it could be accepted that the main function of T cells is to recognize and regulate peptides that are harmful to self-cells. Accordingly, variations in T cell subsets might depend on the origin and structure of pathogenic peptides and possibly biochemicals (identified with TLRs or other receptors of T cells) that exist in disordered conditions.

B cells produce antibodies against pathogenic proteins and have also several subsets. However, B cells are not mere antibody producers; they function as sophisticated immune effectors and modulators by secreting cytokines and presenting antigens within the immune network (84). The existence of “regulatory B cells” that secrete anti-inflammatory cytokines such as IL-10 (85) suggests that B cell subsets also play dynamic roles for the control of pathogenic proteins—and possibly other toxic substances—for cell protection. In general, isolated B cells cannot produce antibodies against the peptides being controlled by T cells, small non-protein molecules such as monoamines and neuropeptides, chemicals, or biochemicals. Among them, some non-protein materials, called haptens, are found in chemicals such as nitrophenols and biochemicals such as polysaccharides and nucleic acids and can bind certain proteins called carriers (86). Various hapten antigens, such as addictive drugs or the constituents of pathogens, and potential carrier proteins have been evaluated for vaccine development (87, 88). The binding of two materials might occur *in vivo* on occasion. Clinical observations in SLE and RA provide robust evidence for hapten-carrier-like interactions. In SLE, double-stranded DNA functions as a hapten when it is complexed with histone proteins (89), whereas in RA, the post-translational modification of proteins (or peptides), changing of arginine to citrulline can create neoepitope antigens that trigger antibody production (90). These phenomena confirm that even endogenous materials such as DNA and transformed proteins can be recategorized as immunogenic substances that induce adaptive immune responses. Specific classically produced antibodies against pathogenic proteins and those against hapten–carrier conjugates play roles in the MHC-restricted immune pathways, where helper T cells play a critical role (91). At the same time, natural antibodies, primarily produced by B-1 cell subsets, provide an immediate and T cell–independent response against non-protein antigens such as bacterial polysaccharides and toxic debris from injured self-cells, functioning as a critical first line of defense like the innate immune response (92).

Although anti-capsid antibodies can bind viruses *in vitro* and possibly *in vivo*, a virion itself is not an effective antigen in diagnostic assays or kits. The efficacy of diagnostic assays depends heavily on the selection of refined antigens defined from infectious and autoimmune diseases (93, 94). Thus, it is possible that nonspecific natural antibodies can bind to biochemicals that are conjugated with proteins on the assays, and that antibodies from a specific B cell clone can react to nonspecific protein antigens through cross-reactivity in *in vitro* studies.

### 4.2 Specific vs. nonspecific clones in immune systems

Cross-reactive T cell clones and antibodies have been found to respond to structurally analogous but distinct proteins or peptides as antigens (95, 96), and cross-reactive or nonspecific immune cell clones can be responsible for disease pathogenesis (97, 98). It should be emphasized that there are antigen-specific clones and non-specific clones, which target antigens, including pathogenic peptides/proteins and toxic non-protein substances. Upon exposure to novel antigens, functionally relevant but nonspecific, pre-existing, or polyreactive clones are recruited first through cross-reactivity, because the generation of pathogen-specific T cell clones and antibodies requires at least several days after the onset of disease symptoms. Activated nonspecific adaptive immune cells, particularly T cells responding to excess antigens bound on target cells, release a range of cytokines and biochemicals. Among them, cytotoxic factors can cause target cell injury through the so-called cytokine storms observed in influenza, COVID-19, septic conditions, and other conditions such as hemophagocytic syndromes, chimeric antigen receptor (CAR)-T cell-associated toxicity, immune checkpoint inhibitor toxicity, and graft-versus-host disease (99, 100). The immune cells involved in various cytokine storms are the same type of immune components in our body. It is possible that the etiological substances and corresponding immune cells of each cytokine storm could differ, but target cell injuries caused by overactivation of the primary immune components with cytokine imbalances occur in any cytokine storms.

These phenomena might occur within the innate immune system, reflecting certain aspects of immunological memory associated with trained immunity. The phenomenon of trained immunity demonstrates that innate immune cells such as NK cells, monocytes, myeloid cells and even self-renewing hematopoietic cells undergo epigenetic reprogramming to develop functionally relevant clones upon exposure to infectious insults (38, 101). Over time, these primed innate immune cells, including granulocytes, might become more specific clones with immunological memory during repeated immunological or biological insults. For example, when novel agents responsible for allergic reactions interact with affinitive receptors on target cells, nonspecific or less-relevant clones of eosinophils, basophils, or mast cells might be activated via cross-reactivity. The activation of those cells results in the release of cytotoxic and inflammatory mediators, including major basic proteins and eosinophilic cationic proteins from eosinophils, and histamines and lipid mediators from mast cells, which could subsequently affect target cells including barrier epithelial cells in asthma (102, 103), like mechanisms of cytopathy caused by nonspecific adaptive immune responses. Materials such as DAMPs and pathogenic peptides/proteins derived from target cells injured by that allergic reaction might induce inflammation through recruiting corresponding immune cells (12). Children have a maturing immune system, and many patients with asthma, atopic dermatitis, and JIA in early childhood show an improvement or long-term remission over time, indicating that the mature of adaptive or innate immune systems could control the insults from the diseases. Patients with allergies such as asthma or atopic dermatitis might have defective immune clones for controlling initial etiological substances or the substances derived from injured target cells (12).

The duality of the immune system, sometimes called the ‘double-edged sword’ of immunity, might be fundamentally understood as a manifestation of the balance between specific and non-specific clones within the PHS hypothesis. Although specific clones ensure the high-affinity neutralization of etiologic substances with minimal self-cell injury, the compensatory recruitment of non-specific clones—often triggered by an excessive load of cross-reactive antigens— could result in uncontrolled cytotoxic cytokine release and cause target cell injury in organs.

### 4.3 The PHS hypothesis

The PHS hypothesis is a theory in “theoretical biomedicine”. It proposes that all diseases involve etiological substances and that they are regulated by the immune systems under control of the PHS. This hypothesis is based on experiments by previously devoted researchers and my insight from clinical studies on various diseases. From the perspective of organicism, the PHS can be regarded as a high-level supervisory system that elucidates how the immune system coordinates its functions to promote the survival of the organism. The PHS hypothesis has been proposed as a potential explanation for the etiology and pathophysiology of disease entities, including infectious conditions such as influenza, COVID-19, and pneumonia/ARDS (9, 104–106); infection-related immune-mediated diseases such as KD and MIS-C (8, 107); organ-specific disorders such as kidney diseases and CNS diseases, including genetic, prion, and Alzheimer’s diseases (10, 11); cancer (10, 13); and allergic and autoimmune diseases such as asthma, atopic dermatitis, and JIA (12, 108). In brief, all diseases involve etiological substances that are linked to disease onset. For the protection of cells, each component of the immune system regulates these substances based on their size and biochemical properties under the regulation of the PHS. The PHS is also responsible, at least in part, for regulating intracellular and systemic protein (or peptide) deficiencies. The adaptive immune system regulates pathogenic peptides and proteins, and the innate immune system manages toxic non-protein substances, as well as larger complex materials such as virions, bacteria, parasites, and apoptotic or necrotic bodies. Immune effectors within the innate immune cells, as well as other currently unidentified immune components or effectors—such as prions and amyloid proteins—are involved in the regulation of toxic non-protein/peptide substances (11, 12) Previously published articles discussed both unresolved questions in each disease and more details of the PHS hypothesis (12). Etiological substances and corresponding immune systems and their components are shown in **Table 1**. Also, a proposed unified mechanism for disease onset and progression within the PHS hypothesis is shown in **Figure 1**.

**Table 1:** Etiological substances and corresponding immune components in disease, according to the PHS hypothesis.

Control systems	Etiological substances/primary target	Main functions
1) Adaptive immune system		
T cells	Pathogenic peptides originated from mainly self-cells and outside complex materials such as pathogens and natural toxins. They exist as free form and bind to receptors of target cells including cancer cells.	Control of pathogenic peptides through secretion of cytokines (possibly peptides) and immune effectors within the cells in the MHC-restricted (TCR-associated) and non-MHC-restricted conditions
B cells	Pathogenic proteins originated from self-cells and complex materials such as pathogens and natural toxins. They exist as free form and bind to receptors of target cells including cancer cells.	Control of pathogenic proteins by secretion of antibodies within cells through neutralization in the MHC-restricted (BCR-associated) and non-MHC-restricted conditions
2) Innate immune system		
Natural killer cells	Transformed cells such as virus-infected cells and tumor cells	Control of target cells through their recognizing receptors and immune effectors within the cells
Tissue macrophage-lineage cells	Complex protein/peptide containing materials such as pathogens for antigen presentation to adaptive immune cells.	Antigen presentation to adaptive immune cells. Possible control of communications between immune cells and affected organ cells including cancers cells in TMEs via cytokine networks.
Phagocytes (neutrophils and circulating monocyte/macrophages)	Large complex materials such as viruses, bacteria, parasites originated from outside, and apoptotic and necrotic bodies originated from self-cells.	Clearance of large pathogenic materials and debris from cell injury caused by infection, trauma and other conditions, through receptors and secretion of immune effectors within the cells.
Mast cells, basophils, eosinophils	Mainly external toxic non-protein substances such as elements, chemicals, and biochemicals in cases of allergy and parasite infection	These cells recognized the substances by receptors of the cells and control the substances through immune effectors within the cells and inflammation involved in these cells (12)
3) Unidentified immune components against toxic non-protein materials	Mainly internal non-protein toxic or inflammation-inducing substances, including elements, monoamins, neuropeptides, RNAs, DNAs, chemicals, and biochemicals	TLRs- or alarmin receptors-associated immune responses, natural antibodies, and immune proteins and/or peptides control these diverse substances. The PrP gene products and amyloid proteins, control pathogenic monoamine or neuropeptide metabolites especially in CNS (11)
4) Systems for production of alternative proteins	A essential protein deficiency or malfunctional protein of the cells in tissues or within a regenerating cell	The systemic and intracellular PHS control in part insults from a protein deficiency or malfunctional protein, which are associated with genetic diseases and cancer (13)

MHC, major histocompatibility complex; TCR, T cell receptor; BCR, B cell receptor; TME, tumor microenvironment; PrP, prion; CNS, central nervous system.

**Figure 1:**
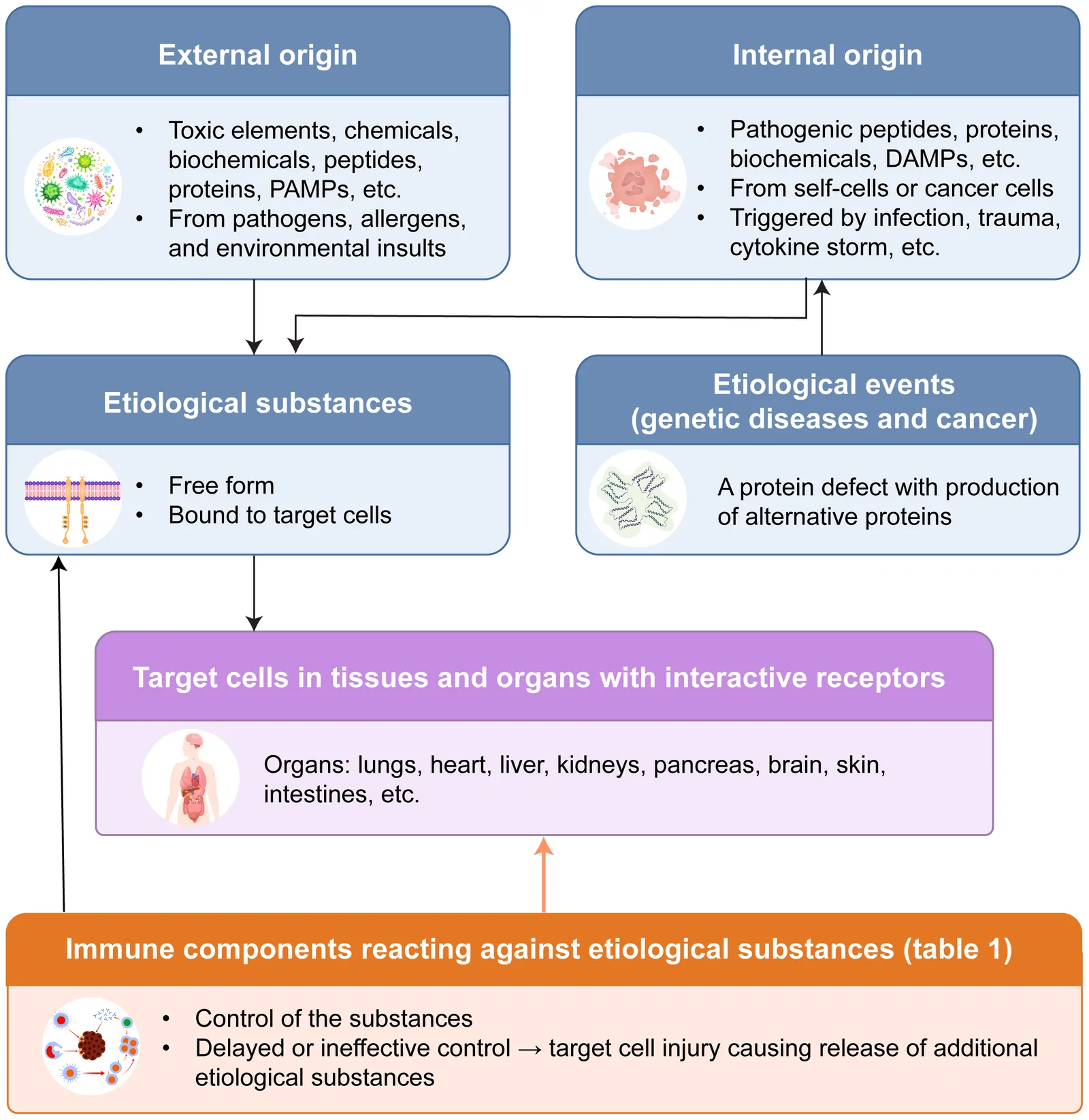
Schematic representation of disease pathogenesis in the PHS hypothesis. The etiological substances of disease pathogenesis are divided into two primary sources: external substances include toxic elements, chemicals, biochemicals, and PAMPs from pathogens, allergens, and environmental insults; internal substances consist of pathogenic peptides and proteins, DAMPs, and biochemicals released from self-cells (or cancer cells on occasion) injured by infection, trauma, or other conditions such as cytokine storms. Etiological substances can exist in free forms and interact with specific receptors on target cells in organs such as the lungs, heart, kidneys, liver, and brain via systemic circulation. The binding of these substances triggers the recruitment of corresponding immune components within the PHS (as detailed in **Table 1**) to the target cells, eliciting inflammation for cell protection. The successful control (or neutralization) of etiological substances by specific immune clones leads to the restoration of systemic homeostasis. Delayed or ineffective control, often involving non-specific immune hyper-reactions, results in target cell injury. This injury triggers the release of additional endogenous etiological substances, creating a pathological feedback loop that drives disease progression. In genetic diseases and cancer, the PHS manages protein deficiencies or dysfunctional proteins in part by triggering the production of alternative proteins, which can eventually lead to systemic imbalances.

### 4.4 Genetic defects of immune components in immune systems

The host’s genetic architecture plays a critical role in shaping both the composition and functional capacity of immune components. Consequently, the specificity of T cell and B cell responses to pathogenic peptide or protein antigens is determined by the genes encoding for recombination of TCRs and BCRs. Thus, it is possible that patients with autoimmune diseases might have certain genetic defects such as lack of genes, including D-V-J genes, for recombination of TCR or BCR to control specific peptides or proteins. In a comparable manner, the expression and activity of other immune components or effectors—innate immune cells such as neutrophils, eosinophils and monocytes, complement proteins, and immune proteins such as prions and amyloidogenic proteins—that can target small toxic molecules are subject to genetic regulation (109, 110). Accordingly, new immunodeficiency syndromes and chronic inflammatory conditions associated with gene defects of immune components have been identified. In these contexts, the autoinflammatory diseases are categorized and include familial mediterranean fever, TNF-receptor-associated periodic syndrome, and cryopyrin-associated periodic syndrome. These diseases are characterized by gene defects in mainly innated immune cells or their signal transduction pathways (111). However, there may be etiological substances responsible for various clinical symptoms and signs in each disease in this disease category, and defective control systems against them may affect disease processes as well as in autoimmune diseases in the PHS hypothesis.

On the other hand, the function of the immune system changes throughout life (112), and therefore the severity and prognosis of infectious and infection-related immune-mediated diseases differ between children, adults, and elderly (9, 108).

## 5 Pathophysiology of diseases through the PHS hypothesis

As indicated in **Table 1**, specific subsets of immune cells regulate toxic and bioactive etiological substances, both in their free form and their form when bound to target cells. In general, immune cells detect toxic substances via receptors expressed on their surfaces and regulate or neutralize those substances through intracellular immune effectors with subsequently occurring inflammatory responses. Lymphocytes and neutrophils are continuously changing in both steady states and disordered conditions. These cells are present in the blood in greater proportions than eosinophils, basophils, and NK cells. Additionally, the neutrophil-to-lymphocyte ratio varies with ages during childhood and fluctuates depending on the stage of various diseases (113). It is reasonable to consider that the proportion of immune cells present in the blood represents the relative functional demand placed on each cell population for maintaining systemic homeostasis. Neutrophils are typically the initial responders, and then lymphocytes appear in any pathological lesions that involve cell injury associated with disease (114). In the human body, cell turnover occurs in both normal physiological states and pathological conditions. Consequently, apoptosis and necrosis are ongoing processes that regularly take place throughout human tissues, and neutrophils and lymphocytes within both immune systems regulate materials that originate from damaged self-cells, such as pathogenic peptides/proteins and apoptotic or necrotic bodies, in both steady-state and pathological conditions. Comparatively, monocytes, NK cells, eosinophils, and basophils (considered mobile forms of mast cells) are present at lower proportions in the blood. This distribution suggests that these cells may be less active in steady states than in pathological conditions. Among the immune components, mast cells, B cell subsets responsible for producing IgA or IgE, and specific subsets of T cells such as γδ-T cells and ILCs are predominantly located at the interface between the host and environment, including the intestines, respiratory tract, skin, and genitourinary tract (12, 115, 116). This distribution suggests that these cells principally may function to manage toxic substances that originate from the external environment.

Through the PHS hypothesis, established immunological phenomena can be reconsidered, which opens novel perspectives on unresolved disease-related challenges.

### 5.1 About infectious and infection-related immune diseases in organs

The microbiota is an interdependent ecological system of microorganisms that evolves with a host species (117). Most human pathogens, including bacteria, viruses, and fungi, originate from strains within the microbiota and exhibit species-specific characteristics (118). Furthermore, recent studies based on genomics have revealed that only parts of strains in microbiota can be detected by current culture methods (119). Occasionally, strains in the microbiota invade the host and establish a *focus*—a localized site where etiological substances are generated and subsequently released into the systemic circulation. The foci in intracellular pathogen infections, such as viral infections, are difficult to detect and could be defined as localized lesions containing the initially infected cells, whereas the foci in extracellular pathogen infections can be defined as the easily seen localized lesions found in appendicitis, osteomyelitis, and periodontitis. For instance, virus-infected cells, the foci, contain replicated virions, byproducts of viruses such as capsid proteins, viral enzymes, nucleic acids (viral DNAs and RNAs) and other PAMPs, and cell-origin substances such as DAMPs, biochemicals, and pathogenic peptides/proteins (120, 121). Among these materials, etiological substances spread and bind to target cells in tissues or organs via systemic circulation, inducing inflammation with corresponding immune components. The injury of target cells during this process caused by the cytokine storm can release intracellular materials and permit the invasion of bacterial strains through broken barriers, eliciting further inflammation. Thus, the incubation period of intracellular infectious diseases can describe the interval between the initial pathogen invasion on host cells and the onset of viremia or bacteremia. Accordingly, viremia or bacteremia indicates not only the dissemination of pathogens but also the spread of various intracellular or extracellular substances that are present in the foci into all tissues via the systemic circulation (122). Certain extracellular bacterial pathogens, including streptococci and *Mycoplasma pneumoniae*, can invade host cells in specific circumstances (123, 124). Therefore, it can be explained that Group A streptococci cause not only scarlet fever but also acute rheumatic fever or acute poststreptococcal glomerulonephritis following primary infection. Similarly, *Mycoplasma pneumoniae* can lead to both pneumonia and extrapulmonary diseases through mechanisms comparable to those observed with other respiratory viruses (125). Bacteria capable of intracellular invasion, including *Mycobacterium* and *Salmonella* species, as well as intracellular pathogens such as rickettsia and chlamydia species, are associated not only with infectious diseases but also with acute and chronic immune-mediated conditions, including autoimmune conditions (126, 127). Given that infectious and infection-related acute and chronic immune-mediated diseases share similar pathophysiological mechanisms under the PHS hypothesis (107), the phenotypes of these diseases, including chronic inflammatory or autoimmune diseases associated with infection and affecting various organs, are determined by the etiological substances derived from the foci, the specific target cells of various organs that have interacting receptors for those substances, and the ability of corresponding immune responses elicited against those substances. Variations in clinical outcomes among individuals are also determined by the burden of etiological substances and the functional capacity of immune components to control them.

### 5.2 About autoimmune and allergic diseases

In the PHS hypothesis, the pathophysiology of autoimmune diseases can be attributed to insufficient immune regulation by improper immune components, which cause sustained activation of non-specific, cross-reactive T cell or B cell clones. Similarly, the activation of non-specific or cross-reactive clones of innate immune cells, such as eosinophils and mast cells, and cross-reactive IgEs are responsible for clinical symptoms in allergies (12). In general, the major etiological substances in autoimmune diseases are pathogenic peptides/proteins that originate from self-cells, whereas those in allergies are non-peptide/protein materials, including elements, chemicals, and biochemicals, that originate from outside the host. However, all components of both immune systems are involved in both entities because target cell injury eventually occurs during disease processes, regardless of the origin of the causative substance.

The prevalence and incidence of the diseases—acute infection-related immune-mediated conditions such as KD and MIS-C; chronic disorders such as JIA, inflammatory bowel disease, Behçet’s disease, RA, and SLE; and allergic diseases such as asthma and atopic dermatitis—vary across ethnic groups and populations. Genetic factors and environmental factors have been introduced to explain this variance (128, 129). The compositions of microbiota differ according to age in childhood and ethnic groups and are influenced by environmental insults introduced by diet and antibiotics (130, 131). If pathogenic strains in the microbiota of other hosts encounter a new host, they first colonize in the host as normal flora and then occasionally invade the host or host cells, making the foci and eliciting the symptoms of acute and chronic infection–related immune-mediated diseases. Thus, the incidence of diseases such as KD and JIA depends on the distribution of pathogenic strains of microbiota in ages in childhood and in ethnic groups or populations (108, 132). Also, the prevalence and incidence of allergic diseases depend on the frequency of exposure to external environmental substances around certain ethnic groups or populations, including natural toxins and biochemicals, pollen, air pollution, and chemicals contained in foods. It was proposed that mast cells in the CNS may play a role in allergic episodes by interacting with peripheral mast cells in a relationship named the “mast cell–associated network” (12).

### 5.3 About prion and neurodegenerative diseases, and amyloidosis

The CNS is an immune-privileged area that contains few T cells and immunoglobulins in both steady conditions and certain pathologies such as prion disease and neurodegenerative disorders (133). The PHS hypothesis suggests that in prion diseases (or transmissible spongiform encephalopathies), the etiological substance might not be the prion itself, but biochemicals smaller than prions, which act as neurotoxins and could originate from certain cells infected with dysbiotic strains of microbiota as the focus (11). The products of the PrP gene (i.e., normal PrP^C^ and its isoform PrP^Sc^, prion) might act as primary immune effectors to control those toxic substances, and prions can transfer the disease to individuals through a biding between etiological substances and the prions that have binding sites consisting of enough β-sheets. Therefore, patients or animals with variant PrP genes experience disease progression due to a lack of specific immune effectors. Similarly, the amyloid proteins found in the pathology of neurodegenerative diseases, such as amyloid beta (Aβ) in Alzheimer’s disease and other amyloidosis, might be immune effectors intended to control toxic substances that are very small and target specific neuronal cells or other specific organ cells. Such immune proteins (or possibly peptides) might control initial etiological substances that originate from initial foci, such as infected cells or external sources, and/or inflammation-inducing substances derived from target cells injured by initial insults. The amyloid aggregates observed in pathology could be associated with the progression of target cell injury, and non-specific or defective immune effectors then accumulate as the corpses of soldiers left on the immunologic battlefield (11). Thus, neurodegenerative diseases could also be regarded as immune-mediated diseases, and immune proteins (or peptides) in the CNS might originate from neuroimmune cells such as microglia or mast cells.

### 5.4 About genetic diseases and cancer

The clinical manifestations of genetic diseases are caused by a deficiency or malfunction in a specific protein (or possibly a peptide) produced by a specific cell group in tissues or organs. There are numerous genetic diseases, and single-gene defects can be divided into structural protein defects (often autosomal dominant) or enzymatic protein defects (often autosomal recessive) (134). Under the control of the PHS, it could be expected that a multifunctional protein defect would trigger the production or use of alternative or compensatory proteins (or peptides) *in vivo* to ensure systemic homeostasis. Thus, clinical phenotypes, progression, and outcomes differ somewhat in familial genetic diseases, depending on how well the alternative proteins (or peptides) replace the missing one. Certain genetic diseases cause progressive cell injury in organs during disease progression, and cell injury is associated with inflammation and more frequent turnover time of cells with the risk of cancer development (135, 136). The accumulation of toxic substances in certain target cells, due to defective proteins or possibly the prolonged activation of alternative proteins, could be associated with host cell injury. Those injured cells can induce sterile inflammation, and in some patients, the continuous activation of non-specific immune components might cause chronic inflammation. Accordingly, pathologic findings in these genetic diseases might show the infiltration of corresponding immune components, including T cells, eosinophils, or immune proteins. It is known that immune modulators such as corticosteroids (Cs) effectively improve disease symptoms or progression in certain genetic diseases, such as muscular dystrophies (137).

The risk of cancer is closely related to the number of tissue cell renewals of stem cells (turnover time) (138). Factors that accelerate that process—such as aging, chronic infection, or physicochemical insults—increase the probability of gene defects during cell replacement (139, 140).

The PHS hypothesis provides different views on established cancer biology or immunology at present, in which mutation-driven cell autonomous oncogenesis and T cell-mediated direct cytotoxicity are main streams. In the PHS hypothesis, cancer might begin with a gene defect in stem cells or regenerating cells to replace older cells. When the cells detect that a protein (or a peptide) essential for survival is missing or defective, the intracellular PHS might trigger the activation of new genes to synthesize alternative proteins that compensate for the absent or dysfunctional one. Over time, additional gene defects or other situations could occur, in that the alternative proteins (or peptides) are demanded to be controlled, so the cell must continuously adapt its control system to manage toxic substances and thus ensure survival and homeostasis. Through repeated processes of new gene activation, including genes used at the stage of embryogenesis, the cell eventually might transform into a complete cancer cell—an independent biosystem capable of self-proliferation that is no longer controlled by the host’s systemic PHS (13). The genetic information in cancer cells, even those originating from the same tissue, are known to differ between individuals with its changing during disease processes, and the treatment response and prognosis also differ among patients (141, 142). The variations might be determined by differences in the initial gene defect and the resulting divergence in intracellular homeostasis systems within each cancer cell. Thus, it is expected that the clinical course and ultimate prognosis are determined by the cancer cells themselves, rather than the immune status of the host (143).

It is also plausible that cancer cells may communicate with host cells, including immune cells, through MHCs and cytokine networks to obtain the assistance they need for survival and growth. The primary function of T cells may be to maintain systemic homeostasis by controlling pathogenic peptides, including those that originate from cancer cells. Since cytotoxic T cells attack tumor cells through MHC-restricted manner, conversely, T cells could protect cancer cells because they express the same MHCs as a marker of self-cells when they are affected by external insults such as infections, trauma, or physicochemical insults. Thus, the PHS hypothesis could provide a new interpretation for the effectiveness of immunotherapies in cancer: immune checkpoint inhibitors might inhibit the communication channels between T cells and cancer cells and block the help that T cells provide to cancer cells, especially at advanced stages (13). CAR-T cells recognize protein antigens, not peptide antigens, expressed on the target cancer cells via the non-MHC-restricted manner and can thus block receptors or signal transduction pathways, especially in acute B cell leukemia (144). However, an infusion of many CAR-T cells can affect the target cells through cross-reactive immunological routes such as a cytokine storm because true cytotoxic T cells should be MHC-restricted and thus be present in small numbers in treated patients (145).

The clinical symptoms and signs of cancer, such as fever, weight loss, autoimmune phenomena, and cachexia, are not caused by the cancer cells themselves, but by various inflammation-inducing substances derived from cancer cells and injured self-cells that drive corresponding immune reactions eliciting inflammation (146). In addition, patients with late-stage cancers show the depletion (or paralysis) of all immune cells, which is also found in the advanced stages of severe infections and other conditions (147), suggesting that at the late stages of disease, an excess of toxic substances might overwhelm the capacity of the host’s immune systems.

## 6 Prospectives for treating disease

Inflammation represents a dynamic interaction between toxic substances and immune responses during disease processes (148). Within the PHS hypothesis, in general, acute inflammation is caused by nonspecific immune clones, together with cytokine imbalances on occasion before the appearance of specific clones, whereas chronic inflammation is partially maintained by nonspecific, functionally relevant immune clones due to a lack or delayed appearance of specific ones. Immune modulators, especially Cs, have been used to treat acute and chronic inflammation in many diseases, including infectious diseases, allergies, cancer, and abnormal tissue repair processes such as keloids. Cs primarily might target the non-specific arm of the immune system. By suppressing non-specific or immature T cells, B cells, and innate components such as eosinophils and mast cells, Cs could induce rapid stabilization of the hyperimmune state through mechanisms, including pro-inflammatory-gene suppression and apoptosis (149). Accordingly, the PHS hypothesis provide a rationale for the early use of immune modulators, especially Cs and intravenous immunoglobulin (IVIG), in patients with severe acute infectious diseases, including influenza, COVID-19, and *Mycoplasma pneumoniae* pneumonia (104, 105, 150); acute infection–related immune-mediated diseases such as KD and MIS-C (8, 107); and progressive acute or subacute whole organ–specific diseases such as ARDS, fulminant hepatitis, extensive cardiomyopathy, rapidly progressive glomerulonephritis, necrotizing pancreatitis, bilateral adrenal necrosis, extensive epidermolysis, rapidly progressive arthropathies, and certain acute encephalopathies, which all have similar pathogeneses according to the PHS hypothesis (106). It has now been proven that among the various therapeutic trials during the COVID-19 pandemic, only Cs reduced the mortality of severe COVID-19 (151), and based on the PHS hypothesis, the author and colleagues recommended preemptive use of Cs for severe cases beginning in the early stage of the pandemic (105, 152). However, immune-modulators, including Cs, IVIG, and biologics, are still used as palliative measures because they cannot control or kill enemies, only control untrained combatants or mislead operations in the battlefield especially at critical stage of the diseases.

The progression and prognosis of diseases, including autoimmune diseases, neurodegenerative diseases, and cancer, might depend on the immune status of the host. However, diseases also produce etiological or inflammation-inducing substances that spur the production of corresponding immune components at each stage of disease progression. Various biological changes have been reported during disease processes, including changed expression of inflammation-associated or tumor-associated genes, epigenetic changes such as microRNA and gene methylation, and changes in immune cells, immune proteins, and metabolic biochemicals that can serve as biomarkers for diagnosis or prognosis. On the other hand, which biomarkers are associated with disease progression, and which are associated with secondary phenomena initiated as immune responses for disease control remain to be proven.

At this time, the etiological substances of disease-onset, their foci, their target cells in organs, and the immune components associated with each disease are not clearly defined. Theoretically, a disease caused by a specific etiological substance that elicits cell injury is needed to study the immune networks in diseases. Although pathogenic peptides within self-cells are the major etiological substances in the PHS hypothesis, it is difficult to identify them among numerous peptides within affected cells. Each cell group in various tissues and organs has distinct peptide pools as the foci and distinct receptors as the target cells. Furthermore, a pathogenic peptide identified in one person might not affect another person because the major receptors of peptides are polymorphic MHCs. Known immune components have diverse subsets and functions, and new functions have been revealed through studies of various disordered conditions. On the other hand, many immune components or effectors against etiological substances remain unidentified. For instance, certain cells in the liver work to detoxify various toxic substances by producing corresponding proteins (enzymes) (153), and the liver is the only organ that can regenerate when its workload is increased, such as after a partial resection, similar to the immune system (154).

By looking at disease through the lens of the PHS hypothesis, physicians could determine treatment strategies not only by understanding the etiology and pathophysiology of the disease but also by understanding the individual patient’s immune status with respect to age, comorbidities, and socioenvironmental factors. With those considerations, management strategies for diseases can take a more holistic approach, beyond the application of recent diagnostic and therapeutic methods that are based on reductionism and whose costs continue to grow over time. In the future, etiological substances and the pathophysiology of each disease will be defined, and thus, proper diagnostic, treatment, and prevention modalities for each disease will also be developed.

## 7 Conclusions

The PHS hypothesis transcends the limitations of current immunological models by providing a unifying principle for the etiology and pathophysiology of human diseases. By shifting the focus from isolated molecular reactions to the integrated regulation of the whole organism, it clarifies why reductionist approaches often fail to identify the primary triggers of diseases, especially chronic and idiopathic illnesses. The PHS is the essence of the living organism as a biosystem and coordinates all subsystems, including the immune system, to maintain systemic homeostasis. Disease onset is driven by a wide spectrum of substances, and small peptides, chemicals, and biochemicals often serve as more critical pathological drivers than the large pathogens or proteins typically studied. The adaptive and innate immune responses are functional tools of the PHS, and their activity reflects the workload required to neutralize specific toxic insults. This paradigm underscores the need for holistic therapeutic interventions. The PHS hypothesis justifies the timely (or preemptive) use of appropriate immune modulators to prevent the irreversible injury of target cells in the lungs and other whole organs caused by nonspecific immune hyper-reactions, as observed in severe COVID-19 and other inflammatory conditions. As we transition toward a more integrated theoretical biomedicine, the PHS hypothesis offers a roadmap for defining the precise etiological substances of diseases whose causes are currently unknown, which will ultimately lead to more effective diagnostic, therapeutic and prevention modalities.
